# A Peptoid-Chelator Selective to Cu^2+^ That Can Extract Copper from Metallothionein-2 and Lead to the Production of ROS

**DOI:** 10.3390/antiox12122031

**Published:** 2023-11-22

**Authors:** Anastasia Esther Behar, Galia Maayan

**Affiliations:** Schulich Faculty of Chemistry, Technion-Israel Institute of Technology Technion City, Haifa 3200008, Israel

**Keywords:** peptoids, copper, chelation, metallothionein-2, reactive oxygen species, anti-cancer therapy

## Abstract

Copper is an essential metal ion that is involved in critical cellular processes, but which can also exhibit toxic effects through its ability to catalyze reactive oxygen species (ROS) formation. Dysregulation of copper homeostasis has been implicated in the progression of several diseases, including cancer. A novel therapeutic approach, extensively studied in recent years, is to capitalize on the increased copper uptake and dependency exhibited by cancer cells and to promote copper-associated ROS production within the tumor microenvironment, leading to the apoptosis of cancer cells. Such an effect can be achieved by selectively chelating copper from copper-bearing metalloproteins in cancer cells, thereby forming a copper–chelator complex that produces ROS and, through this, induces oxidative stress and initiates apoptosis. Herein, we describe a peptoid chelator, **TB**, that is highly suitable to carry this task. Peptoids are N-substituted glycine oligomers that can be efficiently synthesized on a solid support and are also biocompatible; thus, they are considered promising drug candidates. We show, by rigorous spectroscopic techniques, that **TB** is not only selective for Cu(II) ions, but can also effectively extract copper from metallothionein-2, and the formed complex Cu**TB** can promote ROS production. Our findings present a promising first example for the future development of peptoid-based chelators for applications in anti-cancer chelation therapy, highlighting the potential for the prospect of peptoid chelators as therapeutics.

## 1. Introduction

Copper (Cu) is an essential metal ion in numerous cellular functions [1,2] and can serve as an antioxidant within some Cu-containing enzymes such as superoxide dismutase [3,4]. However, Cu can be toxic due to its ability to catalyze the formation of harmful reactive oxygen species (ROS) [5,6]. Disturbances in copper homeostasis have been linked to the development of various diseases, including but not limited to Menkes and Wilson’s disease [7,8], neurodegenerative diseases including Alzheimer’s and Parkinson’s disease [9,10], and cancer [11,12]. Recent studies have reported elevated copper levels in cancer patients [13,14,15], with copper playing a key role in cancer progression by promoting cancer growth [16], angiogenesis [11] and metastasis progression [17]. Therefore, there is a growing interest in developing compounds that target copper for potential anticancer chemotherapies [18].

Current research in this field focuses on two main approaches: Cu chelators, which impede copper-dependent cancer progression by removing copper from the body, and copper ionophores, which modulate and redistribute copper levels, exerting a direct cytotoxic effect on cancer cells [11,18,19]. Implication of copper chelators and ionophores within the tumor microenvironment holds promise against acquired drug resistance, one of the major limitations in current cancer chemotherapy [11]. Notably, well-known copper chelators such as tetrathiomolybdate, trientine, penicillamine, and others, have been already proven to show antitumor efficacy [20,21]. However, many of these compounds still lack specificity, leading to poor tumor selectivity in vivo, and require the administration of higher dosages that often result in severe side effects [18].

The field of Cu metal-binding compounds for cancer treatment is still in its early stages of development [18,19], highlighting the need for selective chelators for copper that will effectively target tumors. Among the various mechanisms of action for copper chelators and ionophores, we were interested in exploring the potential of redistributing excess copper within cancerous cells and promoting intracellular ROS production, ultimately leading to cell apoptosis [22]. To this aim, there is a need to develop an effective chelator that can extract copper from intracellular storage proteins and form a new Cu-chelator complex that can produce ROS by itself and initiate oxidative stress within cancerous cells, while not affecting the Cu-homeostasis and natural antioxidant mechanisms of the normal cells. Of particular interest here is the redistribution of copper stored by metallothioneins (MTs), as they store the excess copper and act as antioxidants to protect cells against copper-related oxidative stress by preventing Cu from redox-cycling and production of ROS [23]. In addition, MTs have been found to play a crucial role in tumor growth, progression, metastasis, and drug resistance [23,24].

While strategies involving Cu-targeting small molecules [25,26,27,28] and Cu-based nanoparticles [29,30,31] have efficiently induced ROS-associated apoptosis mechanisms in various cancer types, they primarily chelate extracellular copper and transport it into cells. In contrast, targeting intracellular copper storage proteins MTs represents a more complex challenge, and redistribution of MT-bound Cu for ROS induction has not been studied before. This is because the potential chelator candidate should not only be able to penetrate the cell but also to compete with the proteins that have a high affinity for copper, criteria that many known agents cannot meet [18].

Peptoids [32], N-substituted glycine oligomers, have gained significant attention in recent years due to their potential applications in various biological processes, including protein interactions [33,34,35] metal binding [36,37], and catalysis [38,39,40,41]. This increased interest is due to the following advantages: (i) their efficient synthesis on a solid support via the “sub-monomer” method [42], which employs primary amines instead of amino acids, eliminating multiple protection/deprotection steps and allowing for the incorporation of different functional side chains, including metal-binding ligands (MBLs); (ii) their ability to adopt well-defined secondary structures when specific bulky-chiral side chains are introduced into their sequences [43,44,45,46,47]; (iii) their good bioavailability being resistant to proteases [48,49], having high membrane permeability [50,51] and tolerance to various conditions such as high temperatures [52], salt concentrations and pH levels [53,54]. Over the past decade, our group has extensively studied peptoids for copper-targeting chelation [43]. We have demonstrated their ability to selectively bind Cu^2+^ at the physiological conditions even in the presence of an excess of other metal ions, with few examples of peptoid-chelators being able to extract Cu from copper-bearing peptides [55]. Moreover, we have recently reported on a peptoid-based chelator that can extract Cu^2+^ from MT-2, while its association constant for Cu^2+^ was rather low [56]. Thus, in this research, we wished to develop a peptoid-chelator that has both high affinity and high selectivity for Cu^2+^, that can extract it from redox-silent MT, and as a result of its extraction, will form a Cu^2+^-peptoid complex that can initiate ROS production.

Capitalizing on our accumulated knowledge and harnessing the peptoids’ advantages, we present here a novel water-soluble helical peptoid chelator that is highly selective for Cu^2+^ ions. We demonstrate the high affinity of this chelator to Cu^2+^ and its ability to compete and extract Cu^2+^ from the Cu-containing protein metallothionein-2. Moreover, we describe the ability of the newly formed Cu-peptoid complex to produce ROS. These results suggest that peptoids represent a highly promising platform for the development of copper-targeting chelators as anticancer agents.

## 2. Materials and Methods

### 2.1. Materials

Rink amide resin was purchased from Novabiochem, Mercury (Rosh Ain, Israel). Trifluoroacetic acid (TFA) and zinc(II) sulfate monohydrate, nickel(II) acetate were purchased from Alfa Aesar, Tzamal (Petach-Tikva, Israel). (S)-(−)-1-phenylethylamine (*N*spe), 4′-chloro-2,2′:6′,2″-terpyridine, piperazine (*N*pip) and manganese(II) acetate tetrahydrate were purchased from Acros Organics, Holland Moran (Yehud, Israel). Copper(II) chloride, cobalt(II) acetate tetrahydrate, bromoacetic acid and chloroacetic acid were purchased from MERCK Millipore, Mercury (Rosh Ain, Israel). 6-bromo-2,2′-bipyridine, 2-methoxyethylamine (*N*me), copper sulfate pentahydrate, N,N′-diisopropylcarbodiimide (DIC), piperidine, HEPES buffer (sodium salt of 2-[4-(2-hydroxyethyl)piperazin-1-yl]ethanesulfonic acid), Sodium ascorbate, acetonitrile (ACN), Methanol (MeOH) and water and HPLC-grade solvents were purchased from Sigma-Aldrich, MERCK, Mercury (Rosh Ain, Israel). Dimethylformamide (DMF) and dichloromethane (DCM) solvents were purchased from Bio-Lab Ltd. (Jerusalem, Israel). Apo-protein metallothionein-2 (MT-2) was bought from Genecust (Boynes, France). All reagents and solvents were used without additional purification. 4′-chloro-2,2′:6′,2″-terpyridine amine (Terpy) and 2-(2,2′-Bipyridine-6-yloxy) ethylamine (Bipy) were synthesized according to previously published procedures [57,58].

### 2.2. Synthesis and Purification of the Peptoid Oligomers

Peptoid oligomers were synthesized manually at room temperature on Rink amide resin using a variation of a previously reported peptoid sub-monomer protocol [42,59]. Typically, 100 mg of resin (0.64 mmol g^−1^) was swollen in dichloromethane (DCM) for 40 min before initiating oligomer synthesis. De-protection of resin was performed by the addition of 20% piperidine solution (0.4 mL in 1.6 mL of Dimethyl formamide (DMF)) and the reaction was allowed to shake at room temperature for 20 min. Piperidine was washed from the resin using DMF (10 mL g^−1^ resin) (3 × 1 min). Bromo-acetylation was completed by adding 20 eq. bromoacetic acid (1.2 M in DMF, 8.5 mL g^−1^ resin, 10.2 mmol g^−1^) and 24 eq. of diisopropylcarbodiimide (DIC) (2 mL g^−1^ resin, 9.4 mmol g^−1^); this reaction was allowed to shake at room temperature for 20 min. Following the reaction, the bromo-acetylation reagents were washed from the resin using DMF (10 mL g^−1^ resin) (3 × 1 min) and 20 eq. of submonomer amine (1.0 M in DMF, 10 mL g^−1^ resin, 10 mmol g^−1^) were added. The amine displacement reaction was allowed to shake at room temperature for 20 min and was followed by multiple washing steps (DMF, 10 mL g^−1^ resin). Bromo-acylations and amine displacement steps were repeated until the desired peptoids were obtained. The incorporation of several amines required alteration of the general protocol by the previously described procedure: Terpy [57], Bipy [58], and *N*pip [60]. To cleave the peptoid oligomers from solid support for analysis, approximately 5 mg of resin was treated with 95% Trifluoroacetic acid (TFA) in water (40 mL g^−1^ resin) for 10 min. The cleavage cocktail was evaporated under nitrogen gas and the peptoid oligomers were re-suspended in 0.5 mL HPLC solvent (1:1 HPLC grade acetonitrile: HPLC-grade water). To cleave the peptoid oligomers from solid support for preparative HPLC, the beads were treated with 5 mL of 95% TFA in water for 45 min. The cleavage cocktail was evaporated under low pressure, re-suspended in 5 mL HPLC solvent, and lyophilized overnight. Peptoid oligomers were analyzed by reversed-phase HPLC (analytical C18 column, 5 μm, 100 Å, 2.0 × 50 mm) on a Jasco UV-2075 or Jasco UV-4075 instrument (Jasco Corporation, Tokyo, Japan) at 214 nm. A linear gradient of 5–95% acetonitrile (ACN) in water (0.1% TFA) over 10 min was used at a flow rate of 0.7 mL min^−1^. Preparative HPLC was performed using a Phenomenex C18 column (15 μm, 100 Å 21.20 × 100 mm) on a Jasco UV-2075 instrument (Jasco Corporation, Tokyo, Japan) at 230 nm. Peaks were eluted with a linear gradient of 5–95% ACN in water (0.1% TFA) over 50 min at a flow rate of 5 mL min^−1^. Mass spectrometry was performed on a Waters LCT Premier mass (Waters Corporation, Arnhem, The Netherlands), Bruker Compass HyStar (Bruker UK Ltd., Coventry, UK), and Advion expression mass (Advion Inc., Ithaca, NY, USA) under electrospray ionization (ESI), direct probe ACN:H_2_O (70:30), flow rate 0.3 mL min^−1^.

### 2.3. General Method for Water Solubility Test

A 1 mg peptoid was taken in an Eppendorf and solvent (water or buffer) was added gradually (e.g., 5 μL per addition) until a clear solution was obtained. The solubility test was repeated three times and average values are presented.

### 2.4. EPR Studies

EPR spectra were taken on a Bruker EMX-10/12 X-band digital spectrometer (Bruker UK Ltd., Coventry, UK) from 2300 G to 4200 G, 3 G amplitude modulation, approximately 9.4 GHz, and at 203 K. Spectra were recorded using a microwave power of 0.64 mW. Samples were prepared in HEPES buffer (50 mM, pH = 7.4) +10% of glycerol (*v*/*v*) as a cryoprotectant. (2,2,6,6-Tetramethyl-1-piperidinyl)oxidanyl (TEMPO, g = 2.0058) was used as a reference for simulations. Spectra simulation and processing were performed with Bruker 32 Bit WIN-EPR Acquisition (version 3.03) and WIN-EPR SimFonia Software (version 1.26 (beta)).

### 2.5. UV/Visible Spectroscopy

In the titration experiments, 3 mL of solvent was first measured as a blank. Then, in a typical experiment, 10 µL of a peptoid solution (5 mM in water) was added (to obtain a 17 µM concentration) and then sequentially titrated with 2–4 µL aliquots of a metal ion solution (5 mM), in multiple steps, until the binding was completed, and the spectrum was measured again. The job plot was determined using UV-Vis spectrometry by varying mole fraction of Cu^2+^ ion and **TB** using 35 μM total molar concentration in un-buffered water solution (pH = 7.0). The absorbance assigned to the formation of Cu**TB** at 330 nm was plotted against the mole fraction χ(Cu^2+^), and the maxima (i.e., change in the plot slope mode) at the χ(Cu^2+^) of ~0.5 was found. The precise value of the mole fraction was determined by interception between the linear curve fitting of the two plot slope modes: χ(Cu^2+^) = 0–0.5 (blue) and χ(Cu^2+^) = 0.5–1 (red). In the selectivity experiment, solutions containing mixtures of metal ions (1 equivalent of Cu(II) ions and 1–20 equivalents of Cu^2+^, Zn^2+^, Co^2+^, Mn^2+^, Ni^2+^) in 3 mL of HEPES buffer (50 mM, pH = 7.4) were first measured as a blank. Then, peptoid was added (10 μL, 5 mM) and the spectrum was measured again. All measurements were performed using an Agilent Cary 60 UV/Vis spectrophotometer (Agilent Technologies, Mulgrave, Melbourne, VIC, Australia), a double-beam, Czerny–Turner monochromator. Data processing was performed with KaleidaGraph software (version 4.5.0).

### 2.6. Synthesis of Metal Complexes for MS Analysis

Samples for MS analysis were prepared shortly before measurements. In a typical experiment, a solution of peptoid oligomers (100–200 µL of 0.05 mM) in un-buffered water or HEPES buffer was treated with a metal solution or mixture of metal solutions, and the mixture was stirred for 30 min prior to MS analysis. Samples from CD experiments were measured as is. Mass spectrometry analysis of the metal complexes was performed on a Waters LCT Premier mass spectrometer (Waters Corporation, Arnhem, The Netherlands), Bruker Compass HyStar (Bruker UK Ltd., Coventry, UK), and Advion expression mass (Advion Inc., Ithaca, NY, USA), direct probe ACN:H_2_O (70:30), flow rate 0.3 mL min^−1^.

### 2.7. Dissociation Constant Calculations

The dissociation constant for Cu^2+^ with peptoid **TB** was measured by using UV-Vis spectroscopy following a competition method. The stock solutions of peptoid **TB**, EDTA, and Cu^2+^ were prepared at 5 mM concentration in water. For EDTA, the pH value was maintained at pH = 7.0. In a competition experiment [61,62], peptoid **TB** and EDTA were taken in a 1:1 ratio at 12 μM and gradually titrated with Cu^2+^ up to 1.2 equivalents. The UV-Vis spectra were monitored in the 200–800 nm range. Following the previously reported method, the slope between ([**TB**]total/[Cu**TB**]-1) and ([EDTA]total/[CuEDTA]-1) was calculated. The slope equals *K_D_*(Cu**TB**) × *K_A_*(CuEDTA) × *α*(EDTA), where *K_D_*(Cu**TB**) is the dissociation constant of Cu**TB**, *K_A_* (CuEDTA) is the association constant of Cu-EDTA complexation and α(EDTA) is the pH correction factor for EDTA. UV measurements were performed using an Agilent Cary 60 UV/Vis spectrophotometer (Agilent Technologies, Mulgrave, Australia), a double-beam, Czerny–Turner monochromator.

### 2.8. Protein Sample Preparation

MT-2 was purchased from Genecust (Boynes, France) in powder form and used without further purification. Prior to every analysis, fresh solutions of protein were prepared by dissolving it in 10 mM HEPES buffer (pH = 7.4), stock concentration—1–2 mg/mL. The exact concentration of MT-2 solution was determined spectrophotometrically from the absorbance at 220 nm (ε_220_ = 48200 M^−1^ cm^−1^) [63] in 0.1 M HCl. The disulfide bonds were reduced by adding aliquots of ×3 times excess of tris(2-carboxyethyl)phosphine (TCEP) solution per 1 thiol group, as TCEP is known to not react with copper. Stock solution of TCEP was prepared at 30 mM concentration in pH = 7.4 10 mM HEPES buffer.

### 2.9. Circular Dichroism Spectroscopy

Approximately 200 µL solutions (5 mM in water) of lyophilized peptoid powder, Cu^2+^ solution, and protein solution were prepared immediately before CD measurements. CD scans were performed at 25 °C at a concentration of 100 µM (for peptoid-only measurements), and 25–200 µM (for protein-peptoid experiments) in a solution of HEPES buffer (10 mM, pH = 7.4). The spectra were obtained by averaging 4 scans per sample in a fused quartz cell (path length = 0.1 cm). Scans were performed over the 370 to 190 nm region using a 50 nm min^−1^ scan rate. CD measurements were performed using a circular dichroism spectrometer Applied Photophysics Chirascan (Applied Photophysics Ltd., Leatherhead, UK). Data processing was performed with KaleidaGraph software (version 4.5.0).

### 2.10. UV/Visible Spectrophotometry for Kinetics and Kinetics of ROS Formation

Reagents, except the peptoid and protein, were commercially available and were used as received. All the solutions were prepared in milliQ water (resistance: 18.2 MΩ) immediately before experiments. A stock solution of HEPES buffer was prepared at 500 mm, pH = 7.4, and then diluted to 10 mM. Sodium ascorbate was prepared at 5 mm each day because of the quick degradation of the ascorbate. All pH values are given with a ±0.2 pH unit error. UV/Vis kinetics and ROS kinetic experiments were recorded with a spectrophotometer Agilent Cary 60 UV/Vis spectrophotometer (Agilent Technologies, Mulgrave, Australia), a double-beam, Czerny–Turner monochromator with external stirrer of cuvette holder, in 1 cm path-length quartz cuvettes, with 800 rpm stirring. The samples were prepared from stock solutions of MT-2, **TB**, Cu^2+^, diluted to 1.6, 10, and 9 µM, respectively, in 10 mM HEPES solution, pH = 7.4. Ascorbate was diluted to 100 µM.

## 3. Results and Discussion

### 3.1. Peptoid Oligomer Design

**Figure 1:**
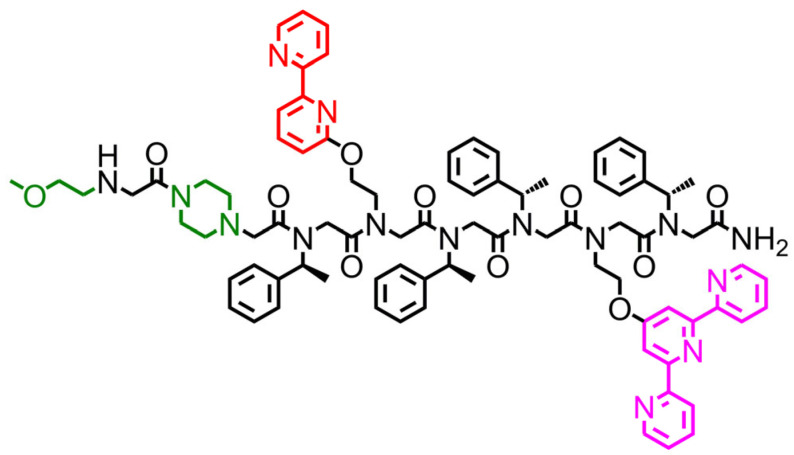
Chemical structure of **TB**, the peptoid oligomer used in this study.

The peptoid **TB** (Figure 1) was designed to be both helical and water-soluble. We previously showed that the peptoid’s helicity enables pre-organization of metal-binding ligands [64], which is required for selective binding of Cu [64]. Based on the relevant literature, we chose to incorporate (S)-(−)-1-phenylethylamine (*N*spe) side chains that are considered helix-inducers [52] and pre-organize two metal-binding ligands within helical **TB** at positions *i* and *i*+3 such that they face the same site of the helix to secure the strong intramolecular binding. As the metal-binding ligands, we decided to incorporate 2,2′:6′,2″-Terpyridine (Terpy) and 2,2′-bipyridine (Bipy) groups. Bipy and Terpy ligands were chosen because they are bi- and tridentate ligands that can flip between the *cis* and *trans* conformations of N-atoms at different pH conditions, as a result of steric disturbance or upon binding to metal ions [65]. In addition, we have recently shown that Bipy ligands within the peptoid-based helical chelator can dissociate into a hypodentatic mode when binding Cu(I) if a more appropriate coordination environment is provided [66]. Therefore, we anticipate, that the relative flexibility of the Bipy-Terpy coordination environment about the metal center will lead to the binding of Cu in an adjustable coordination geometry that will enable high affinity and selectivity, while allowing the redox activity of the Cu-center to catalyze ROS production. Finally, in order to provide solubility of the peptoid’s hydrophobic sequence in aqueous solutions, we chose to incorporate one piperazine moiety at the N-terminus [60] and cap it to prevent its involvement in metal binding, which can decrease the selectivity to Cu [55]. To this aim, we choose to add a 2-methoxyethylamine (*N*me) [57] group at the N-terminus (after the incorporation of piperazine). The *N*me group is hydrophilic and achiral; thus, it can improve the water-solubility of **TB** (compensating for any potential decrease in solubility due to capping of the secondary amine of piperazine) without affecting the peptoid’s helical structure.

### 3.2. Characterisation of CuTB Binding

**Figure 2:**
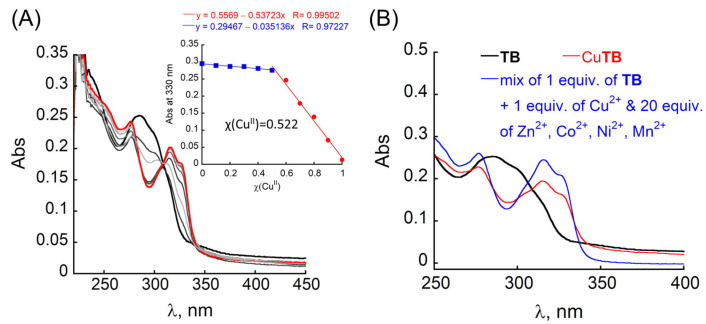
(**A**) UV/Vis titration of **TB** (10 μM) with Cu^2+^ ions in HEPES buffer (50 mM, pH = 7.4): 1 equiv. of metal-free **TB** (black), gradual addition of 0.2 μL of Cu^2+^/per step (grey), and final UV/Vis spectrum of formed CuTB complex (red). Inset: Job plot of **TB** with Cu^2+^ (35 μM total concentration). (**B**) UV/Vis spectra of **TB** (17 μM), its Cu^2+^ and Zn complexes, and the complexes formed upon the addition of a mixture of 1 equiv. of Cu^2+^ and 20 equiv. of each Zn^2+^, Co^2+^, Mn^2+^ and Ni^2+^.

Peptoid octamer **TB** was synthesized via the sub-monomer method on solid support, purified by preparative HPLC, and characterized by analytical HPLC and ESI-MS (Figures S1 and S2). The molecular weight measured by ESI-MS was consistent with the expected mass. First, the solubility of **TB** was evaluated by a previously reported method [60]. As anticipated, **TB** showed high solubility in both un-buffered water (2.70 × 10^5^ mg mL^−1^ at pH = 7.0) and HEPES buffer (2.24 × 10^5^ mg mL^−1^ at pH = 7.4). Next, we studied the binding of **TB** to Cu^2+^ by UV/Vis spectroscopy. The UV/Vis spectrum of metal-free **TB** in both un-buffered water HEPES buffer (50 mM, pH = 7.4), exhibits a broad absorption band at λ = 285 and 300 nm, arising from the overlap of the signals from Terpy and Bipy ligands, respectively (Figure 2A, black). The addition of Cu^2+^ (using CuCl_2_ as a precursor) resulted in the disappearance of these two bands and the formation of a new band near λ = 276 as well as a broad double-maxima band with λ_max_ = 316 nm and 330 nm, respectively (Figure 2A). The metal-to-peptoid ratio plot constructed from this titration suggested the formation of a 1:1 intramolecular Cu-peptoid complex (Figure S3A, inset), and simultaneous binding of Cu^2+^ to both Terpy and Bipy ligands. To further support this suggested stoichiometry of the Cu**TB** complex, we conducted a Job plot experiment. The total molar concentration of a mixture solution containing both Cu^2+^ and **TB** was kept constant at 35 μM, while their mole fractions (χ) were varied, and the UV/Vis spectra of these mixtures were recorded. The absorbance proportional to Cu**TB** complex formation at 330 nm was plotted against the mole fraction (Figure 2A, inset), and from the intersection point at χ = 0.522, a stoichiometry ratio was determined to be 1.09 [66], supporting the metal-to-peptoid ratio obtained from the UV/Vis titrations.

In addition to UV/Vis experiments, the stoichiometric ratio was confirmed by HR-MS techniques: 1 equiv. of **TB** was mixed with 1 equiv. of Cu^2+^ in HEPES buffer (50 mM, pH = 7.4), and the solution was analyzed by HR-MS. The obtained mass of 1589.7378 *m*/*z* matched the calculated mass of the intramolecular 1:1 [Cu**TB** + Cl^−^] complex (*m*/*z* = 1589.6329, Figure S5), suggesting coordination of chlorine counter ion to Cu center, and isotopic distribution comparison further confirmed the identity of the [Cu**TB** + Cl^−^] species (Figure S6). Notably, HR-MS analysis showed no evidence for the formation of higher-order complexes (e.g., 1:2 or 2:2 complexes, Figure S5). Finally, Cu binding by **TB** was studied by EPR spectroscopy. The X-band EPR measurements were performed in a frozen buffer solution, and the resulting experimental spectrum was simulated to obtain the Hamiltonian parameter as *g*_II_ = 2.25; *g*_┴_ = 2.06 and *A*_II_ = 170 G. These parameters are consistent with the axial environment of Cu-center, suggesting that Cu**TB** adopts a square pyramidal geometry about the copper center [67,68,69] (Figure S7).

The binding affinity of **TB** to Cu^2+^ was estimated by a competition experiment with EDTA [61,62]. The mixture of **TB** and EDTA (12 μM each, in water, at pH = 7.0) was titrated with Cu^2+^ followed by UV/Vis spectroscopy. The obtained data were analyzed according to a previously reported method [62]. The dissociation constant *K*_D_(Cu**TB**) is represented by the slope between ([**TB**]_total_/[Cu**TB**]-1) and ([EDTA]_total_/[CuEDTA]-1), and was found to be 6.28 × 10^−16^ M (Figure S8). From this, we calculated that the association constant *K_A_*(Cu**TB**) is 1.59 × 10^15^ M^−1^. This value reflects a strong binding affinity **[55]** supporting our peptoid design.

We next wished to determine if **TB** is highly selective for Cu^2+^ in the presence of an excess of other metal ions**.** For this purpose, we first conducted UV/Vis titrations of **TB** with other biologically relevant metal ions at the same conditions. **TB** binds Zn^2+^ and Co^2+^, forming 1:1 intramolecular complexes (as suggested from the metal-to-peptoid ratio plots constructed from these titrations, Figure S4A,B), does not bind Mn^2+^ (Figure S4C) and weakly binds to Ni^2+^ (no saturation achieved after addition of up to 5 equiv. of Ni^2+^, Figure S4D). Next, we performed selectivity experiments using UV/Vis measurements: 1 equiv. of **TB** was mixed with a solution containing 1 equiv. of Cu^2+^ and *n* equiv. of each Zn^2+^, Co^2+^, Mn^2+,^ and Ni^2+^ (*n* = 1–20) in HEPES buffer (50 mM, pH = 7.4) and stirred for 5 min before recording a UV/Vis spectrum of this solution. The obtained UV-Vis spectra of mixture solutions resembled the spectrum of Cu**TB** only, suggesting that **TB** can selectively bind Cu^2+^ ions in excess of up to 20 equiv. of other metal ions (Figure S9 and Figure 2B) Furthermore, the HR-MS analysis of the mixture containing 1 equiv. of **TB**, 1 equiv. of Cu^2+^ and 20 equiv. of each Zn^2+^, Co^2+^, Mn^2+,^ and Ni^2+^ showed the mass of exclusively Cu**TB** complex (half-mass *m*/*z* = 807.7972, and isotopic distribution of this complex matched the predicted pattern of the half-mass of [Cu**TB** + acetate] complex (Appendix A and Figures S10–S12).

Overall, these results suggested that **TB** has a high affinity and selectivity for Cu^2+^ ions. Our next goal was to determine whether **TB** can extract Cu from a Cu-containing protein followed by ROS production from Cu**TB**, towards its application in re-distributing Cu from CuMTs. To meet this goal, we set to demonstrate the following: (i) the ability of Cu**TB** to extract Cu from a Cu-containing protein that is present within cells, and (ii) that the Cu**TB** complex can produce ROS.

### 3.3. Extraction of Cu^2+^ from the Cu-Containing Protein Metallothioneine by TB

To demonstrate the ability of **TB** to extract Cu from a Cu-containing protein, we decided to focus on metallothionein-2 (MT-2) and use Circular Dichroism (CD) as our spectroscopic tool for detecting Cu extraction and binding via structural changes. We have commercially obtained MT-2 (apo-protein) and used it for further analysis.

**Figure 3:**
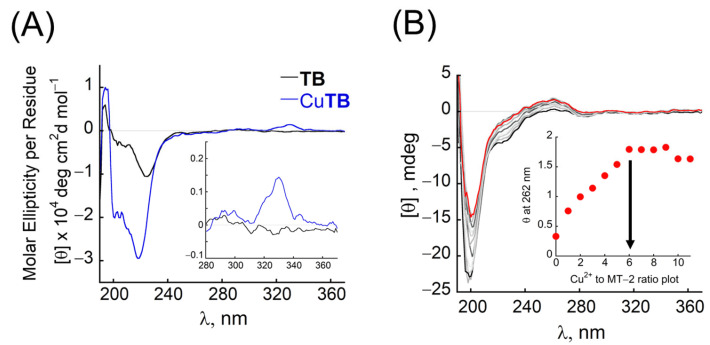
(**A**) CD spectra of metal-free **TB** (100 μM, black) and its Cu^2+^ complex (blue) in HEPES (pH = 7.4, 10 mM). Inset: zoomed CD spectra in the near UV range. (**B**) CD titration of MT-2 protein (25 μM) with Cu^2+^ in HEPES (pH = 7.4, 10 mM, with an excess of TCEP). Inset: Cu^2+^ to protein ratio plot constructed from the CD titration.

Metal-free **TB** exhibits characteristic double-minima, with a minimum at 220 nm that is more pronounced than the additional minimum at 205 nm, indicating the peptoid’s folding into a helical structure in HEPES buffer (pH = 7.4, 10 mM) (Figure 3A, black). Upon addition of 1 equiv. of Cu^2+^, the intensity of this double-minima increases (Figure 3A, blue), and a positive band with a maximum near 330 nm is obtained, corresponding to the π-π* transition of Terpy and Bipy ligands, caused by the interaction between these two chromophores, bound to the same backbone, upon metal coordination (Figure 3A, inset). In contrast, the CD spectrum of metal-free MT-2 at the same conditions exhibits a minimum at 200 nm with a shoulder band near 230 nm (Figure 3B, black). Upon gradual addition of Cu^2+^, the intensity of both bands is decreased until the shoulder band disappears, and a new exciton couplet, comprised of a positive broad peak at 262 nm and a negative broad signal at 286 nm, appeared, with an isodichroic point near 274 nm (Figure 3B, grey and red). Metal-to-protein ratio plot at 262 nm suggests the binding of 6 equiv. of Cu^2+^ (Figure 3B, inset), consistent with the relevant literature [70]. As the addition of Cu^2+^ leads to a decrease in the absorbance band near 200 nm, extraction of Cu^2+^ from CuMT-2 by **TB** should lead to an increase in the intensity of this band. However, as described above, **TB** and Cu**TB** show an intense CD signal in this region, thus the possible overlapping of signals is anticipated to hamper the observation of Cu^2+^ extraction based on this band only. Alternatively, MT-2, CuMT-2, and **TB** do not absorb at the 300–350 nm range, while Cu**TB** produces a single positive band in this region. We therefore anticipated that the extraction of Cu^2+^ from MT-2 would be indicated by the disappearance of the exciton couplet with the positive band at 262 nm upon the addition of **TB** to CuMT-2, together with the appearance of a positive band at 330 nm, indicating the formation of Cu**TB**.

**Figure 4:**
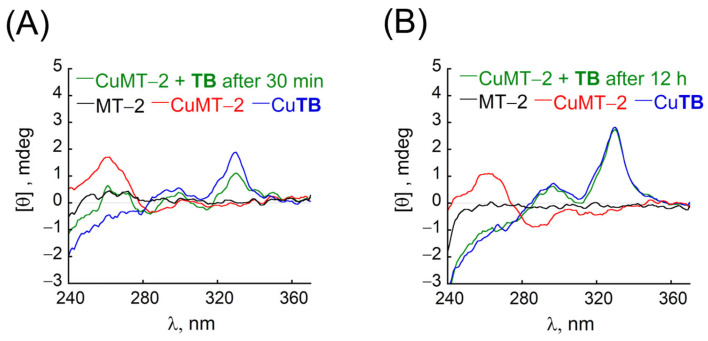
CD studies for Cu^2+^ extraction from CuMT-2 complex in HEPES (pH = 7.4, 10 mM). CD spectrum of the near UV range for free MT-2 (black) and Cu^2+^+MT-2 (red), Cu**TB** (blue), and a mixture of MT-2 + Cu^2+^ + **TB** at (**A**) 30 min or (**B**) 12 h after addition of **TB** (green). Conditions for (**A**): [MT-2] = 25 μM, [Cu^2+^] = [**TB**] = 150 μM; for (**B**): [MT-2] = 33 μM, [Cu^2+^] = [**TB**] = 200 μM.

Accordingly, extraction of Cu^2+^ from CuMT-2 complex by **TB** was studied by CD spectroscopy. First, a mixture of 1 equiv. MT-2 and 6 equiv. of Cu^2+^ in HEPES buffer (10 mM, pH = 7.4 and excess of TCEP) was stirred for 5 min before its CD spectrum was recorded (Figure 4A, black and red). Next, an equimolar amount of **TB** was added to the mixture, and the solution was incubated for 30 min before its CD spectrum was measured and compared to the spectrum of **TB** and Cu^2+^, taken in the same conditions (Figure 4A, green). Compared to CuMT-2, the obtained spectrum showed a decrease in the maximum near 262 nm, and the appearance of the positive band at 330 nm, indicating partial formation of Cu**TB** only 30 min after addition to CuMT-2 mixture (Figure 4A).

Full formation of Cu**TB** was obtained 12 h after the addition of **TB** to CuMT-2, as the obtained CD spectra are identical to the CD spectrum of Cu**TB** in the same conditions, suggesting full extraction of Cu^2+^ from CuMT-2 complex (Figure 4B). Extraction of Cu^2+^ from CuMT-2 complex was further confirmed by ESI-MS of the studied mixture solutions. In addition to peaks assigned to MT-2 (*m*/*z* 476, 736, 974, and 1190), the ESI-MS spectra of the mixture of CuMT-2 taken 30 min after the addition of **TB** showed a peak corresponding to Cu**TB** (*m*/*z* 1588.6) together with a peak corresponding to the free **TB** (*m*/*z* 1489.5, Figure S13C). The ESI-MS spectrum of this mixture taken 12 h after the addition of **TB**, showed, in addition to peaks corresponding to MT-2, only the peak at 1588.5 *m*/*z* without other peaks that could be assigned to **TB (**Figure S13D), suggesting full extraction of Cu from CuMT-2 and exclusive formation of Cu**TB** (for details on MT-2 stability see Appendix B).

**Figure 5:**
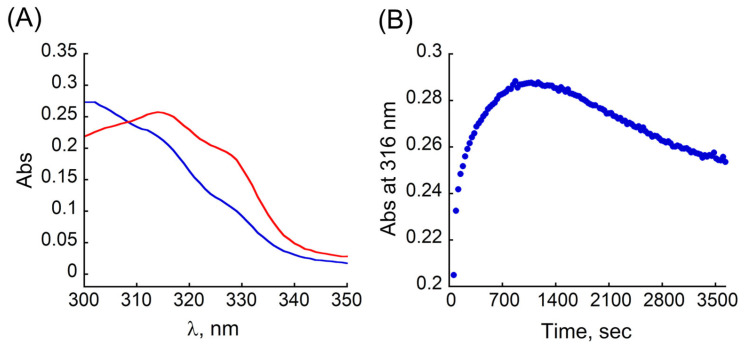
(**A**) UV/Vis spectra in the near UV range of the kinetics of Cu^2+^ extraction from CuMT-2 by **TB**. CuMT-2 + **TB** 0 s after addition (blue) and 3600 s after addition (red). (**B**) Changes in the absorbance at 316 nm of the mixture of CuMT-2 + **TB** as a function of time. Conditions: [MT-2] = 1.6 μM, [Cu^2+^] = 9 μM, [**TB**] = 10 μM, in HEPES buffer (10 mM, pH = 7.4 with an excess of TCEP).

Overall, the CD spectroscopy together with ESI-MS illustrates the ability of **TB** to successfully extract Cu from the CuMT-2 complex, with the full extraction being a thermodynamic process. To further explore the interaction of **TB** with CuMT-2, we set out to evaluate the kinetics of this extraction process. To this aim, we measured the rate of Cu^2+^ binding to **TB** in the presence of MT-2. Thus, quasi-stoichiometric amounts of MT-2 and Cu^2+^ were allowed to react for 500 s to ensure complex formation, followed by the addition of **TB**. The reactions were followed by UV/Vis spectroscopy as depicted in Figure 5A and Figure S15, while Figure 5B represents changes in the absorbance at λ_max_ = 316 nm as a function of time. The spectrum recorded immediately after the addition of **TB** to CuMT-2 (Figure 5A, blue) is similar to that of **TB**, indicating that at time 0 s after the addition of **TB**, Cu**TB** is not formed. Following the changes in the absorbance spectra depicted in Figure 5A and Figure S15, a red shift and increase in intensity of the broad peak at 320 nm is observed, which reaches its maximum about 1000 s after the addition of **TB** (Figure 5B). Thereafter, this band started to decrease, and roughly ~1800 s after the addition of **TB** the shape of the spectrum in this region started to resemble the characteristic double-maxima of Cu**TB** (Figure S15A, green). Finally, about one hour after the addition of **TB**, the UV/Vis spectrum of the mixture resembles the spectrum of Cu**TB** (Figure 5A, red). Overall, as suggested by the CD experiments, the kinetic study confirms that the extraction of Cu^2+^ from the CuMT-2 complex coupled with the formation of Cu**TB** is a thermodynamic process.

### 3.4. Re-Distribution of Cu^2+^ from CuMT2 by TB and ROS Production: A Proof of the Concept

The ability of the Cu**TB** complex to produce ROS was studied by the kinetics of ascorbic consumption assay [71,72,73]. In the presence of both, dioxygen and ascorbate, unbound copper rapidly consumes ascorbate producing the ROS H_2_O_2_ or HO**·**, cycling between its +1 and +2 oxidation state [71,73]. However, Cu bound to MT is redox silent [74,75]. Therefore, any decrease in ascorbate absorption after the addition of a chelator to CuMT-2 will indicate consumption of ascorbate, i.e., ROS production, that is a result of extracting Cu from CuMT-2 and the formation of a redox-active Cu-complex.

**Figure 6:**
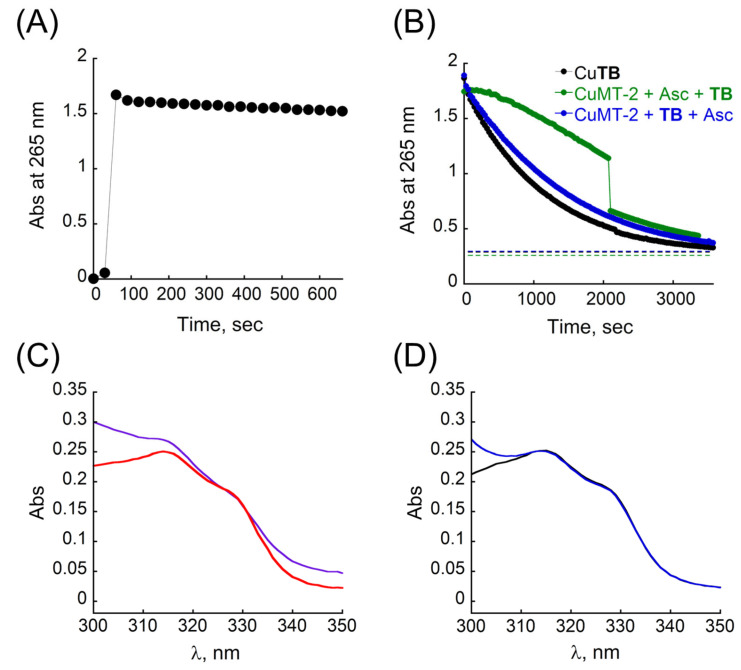
Kinetics of ascorbate consumption, followed by UV/Vis at 265 nm for (**A**) MT-2 + Cu^2+^ + Asc (**B**) **TB** + Cu^2+^ + Asc (black), MT-2 + Cu^2+^ + Asc + **TB** (green), MT-2 + Cu^2+^ + **TB** (1 h) + Asc (blue). The order of components in the text (**A**,**B**) represents the order of addition of the components in the cuvette. The dotted lines in (**B**) correspond to absorbance at 265 nm prior to the addition of Asc for each experiment. (**C**,**D**) UV-Vis spectra of the kinetics of ascorbic consumption experiments for (**C**) MT-2 + Cu^2+^ + Asc + **TB** at 2070 s (purple) and at 2100 s (red) after the addition of **TB**. (**D**) MT-2 + Cu^2+^ + **TB** preincubated for 1 h (black) and at 0 s after the addition of Asc (blue) Conditions: [MT-2] = 1.6 μM, [Cu^2+^] = 9 μM [**TB**] = 10 μM, [Asc] = 100 μM, in HEPES buffer 10 mM pH = 7.4.

First, we investigated whether Cu**TB** can produce ROS by itself. In a typical experiment, 1 equiv. of **TB** was mixed with 0.9 equiv. of Cu^2+^ in HEPES buffer (10 mM, pH = 7.4) in a quartz cuvette under aerobic conditions, and after the formation of the metallopeptoid was confirmed by the stability of its UV/Vis spectrum, sodium ascorbate (Asc) was added to the cuvette and the changes in the absorbance at 265 nm were monitored by UV/Vis (ε = 14,500 M^−1^ cm^−1^) [71,73]. For CuMT-2, there are no changes in the absorbance at 265 nm after addition of Asc to the sample cuvette, indicating that CuMT-2 does not consume Asc and as expected, is redox silent under these experimental conditions (Figure 6A). In contrast, Cu**TB** produces ROS as indicated by a rapid decay of the absorbance of Asc at 265 nm, indicating that Cu**TB** catalyzes the Cu-redox cycle and consumes Asc, reaching full consumption after about 3000 s (Figure 6B, black).

As the extraction of Cu^2+^ from CuMT-2 by **TB** is a thermodynamic process, we wished to compare the ROS production activity profile in two different conditions: (i) when **TB** is added to a mixture solution containing CuMT-2 and Asc, and (ii) when Asc is added to a mixture solution of CuMT-2 and **TB** that were preincubated together for 1 h. In the first conditions, the addition of **TB** to a mixture of CuMT-2 and Asc led to an increase in the absorbance at 265 nm (Figure S16A, black), which is attributed to the absorbance of **TB** itself at 265 nm (see Figure 2A). For the first 5 min, there are no changes in the absorbance at 265 nm (Figure S16A, green), and this correlates well with the slight red shift of the Bipy-Terpy absorbance band and the overall absence of the characteristic shape corresponding to the UV/Vis spectrum of Cu**TB** near 320 nm, indicating that Cu^2+^ is still bound to MT-2 and therefore redox silent (Figure S16A, inset, green). After 10 min, the broad band near 320 nm is more red-shifted and the characteristic shape corresponding to the UV/Vis spectrum of Cu**TB** near 320 nm is starting to form, indicating the initial binding of Cu^2+^ by **TB**. In parallel, there is a small decrease in the absorbance at 265 nm (Figure S16A, light grey), indicating the initial consumption of Asc and the Cu-redox cycle. The slow decay of the absorbance band at 265 nm continues with time, indicating the formation of a small amount of ROS. This could be attributed to either a partial formation of Cu**TB** or to the formation of MT-2–Cu–**TB** ternary species, that have a redox activity and can produce ROS on a small scale. Between 34.5 and 35 min after the addition of **TB** (2070 and 2100 s, respectively), the characteristic double-maxima appears at 316 and 330 nm (Figure 6C, purple and red), indicating the formation of Cu**TB**, and simultaneously, the absorbance at 265 nm decreases rapidly (Figure S16A, purple and red). The slope of the kinetics of ascorbic consumption thereafter is similar to the one observed with Cu**TB** only (similar slope, Figure 6B, green and black). Overall, this experiment demonstrates, one more time, that extraction of Cu^2+^ from CuMT-2 is not a kinetic process, and that it is accompanied by a slow increase in the rate of ROS production. This could be related to the fact that CuMT-2 folds into a multimetallic cluster, which hampers the direct interaction between the **TB** and Cu^2+^ bound to MT-2. When Cu**TB** is fully formed, the production of ROS is rapid and comparable to the ROS production activity of Cu**TB**, which was generated independently (in the absence of MT-2).

In the second conditions, when **TB** is preincubated with CuMT-2 for one hour prior to Asc addition, the corresponding UV/Vis spectra showed that Cu**TB** is fully formed prior to the addition of Asc (Figure 6D, black). The addition of Asc to the mixture (Figure 6D and Figure S16B, blue) initiates the redox cycle, ROS production proceeds and the kinetics of ascorbic consumption is almost identical (similar slope) to the kinetics of ascorbic consumption observed with Cu**TB**, which was generated independently (Figure 6B, blue and black). These results support the observation that the production of ROS is directly dependent on the formation of Cu**TB**, and on its presence in the solution of CuMT-2.

## 4. Conclusions

Herein, we describe a promising first example of a peptoid-based chelator, **TB**, potentially applicable as a copper ionophore. By combining two features in one peptoid chelator, namely, (i) pre-organization of the two metal-binding ligands Bipy and Terpy such that they face the same side of the peptoid helix and (ii) placing piperazine and 2-methoxyethyl amine, which are water-solubilizing groups, at the N-terminus of the peptoid oligomer, resulted in the water-soluble peptoid **TB** having a unique high-affinity and selectivity towards Cu^2+^ at physiological conditions, even in the presence of up to 20 equiv. of other biologically relevant metal ions. We also showed that the extraction of Cu^2+^ from Cu-containing metalloprotein by **TB** leads to ROS production by the newly formed Cu**TB** complex. This has the potential to eventually cause oxidative stress and apoptosis in cancer cells. The current study is the first exploration attempt to generate a peptoid-based drug candidate for possible anti-cancer therapy, and the ability of **TB** to selectively target cancerous cells over normal healthy cells has yet to be explored. Future studies should be focused on optimizing the sequence of **TB** and on in vitro and in vivo studies using **TB** and its modifications; the selectivity of **TB** for cancerous cells over normal cells, which is essential for cancer treatment, could be achieved by applying one of the strategies currently explored in the field of targeted delivery [76], i.e., nano-formulation [77], glucoconjugation [78], and others [79]. Considering peptoids’ bioavailability, this example highlights the potential for future development of peptoid chelators for anti-cancer chelation therapy.

## Data Availability

The data presented in this study are available on request from the corresponding author.

## Supplementary Materials

The following supporting information can be downloaded at https://www.mdpi.com/article/10.3390/antiox12122031/s1. Figure S1: ESI-MS spectra of peptoid **TB**; Figure S2: Analytical HPLC spectra of peptoid **TB**; Figure S3: UV/Vis titration of **TB** with Cu^2+^; Figure S4: UV-Vis titration of **TB** with other metal ions; Figures S5 and S6: HR ESI-MS data for Cu^2+^ complex with **TB**; Figure S7: EPR data; Figure S8: Binding affinity determination by competition method with EDTA; Figures S9–S12: Selectivity studies by UV/Vis and ESI-MS; Figures S13–S15: Supporting ESI-MS, UV/Vis and CD spectra of Cu^2+^ extraction from copper containing protein metallothioneine-2 by **TB**. Figure S16: Full UV/Vis spectra for kinetics of ascorbic consumption experiments. Additional references cited within the Supplementary Materials [61,62,80].

## Appendix A

Coordination of the acetate ion to the Cu-center is plausible as acetate-containing precursor salts were used for the ion source of nickel(II), cobalt(II), and manganese(II) within the frame of selectivity experiments (see Section 2.1). The isotopic distribution of the peak (*m*/*z* = 807.7972) from the mixture studied in a selectivity experiment with 20 equiv. of other metal ions, matched the isotopic distribution of the peak assigned to the half-mass of the Cu**TB** complex, obtained by mixing **TB** and copper (II) acetate as the Cu^2+^ ion source, in the absence of any other metal ions (Figures S11 and S12). Notably, a detailed analysis of the isotopic distribution patterns in the HR-MS spectrum of the mixture was studied in a selectivity experiment with 20 equiv. of other metal ions showed no evidence for the formation of complexes of **TB** with other metal ions (Figure S10).

## Appendix B

MT-2 is known to be stable for a limited time frame in solutions due to the possible oxidation of thiol groups of its Cysteine moieties. The stability of MT-2 during long CD experiments was monitored by ESI-MS (Figure S13C,D) and CD (Figure S14). The ESI-MS spectra show the presence of MT-2. The CD spectra, obtained by subtraction of the Cu**TB** signal from the CD spectra of corresponding mixture experiments, show the presence of protein. For the mixture recorded 30 min after the addition of **TB**, the spectrum resembles the shape of MT-2 partially bound to Cu^2+^ with minima at 208 nm, for the mixture recorded 12 h after the shape of CD spectrum resembles MT-2 with minima shifted to 213 nm, probably due to lose of protein structure upon full extraction of Cu^2+^.
