# Supplementary material for: A Peptoid-Chelator Selective to Cu2+ That Can Extract Copper from Metallothionein-2 and Lead to the Production of ROS
Source: Antioxidants (Basel). 2023 Nov 22;12(12):2031. doi: 10.3390/antiox12122031 (PMC10741037; doi:10.3390/antiox12122031)
Supplement: Supplementary file 1 [file antioxidants-12-02031-s001.zip › antioxidants-2688963-supplementary.pdf]

Supporting Information for:

A Peptoid-Chelator Selective to Cu^{2+} that can Extract Copper from Metallothionein-2 and Lead to the Production of ROS

Anastasia E. Behar [1], and Galia Maayan [1,*]

[1] Schulich Faculty of Chemistry, Technion-Israel Institute of Technology Technion City, Haifa 3200008, Israel
* Correspondence: gm92@technion.ac.il

Table of contents:

ESI-MS and HPLC data of peptoid oligomer **TB** Page 2
UV-Vis titrations with metals Page 3
HR ESI-MS data for Cu^{2+} complex with **TB** Page 4
EPR data Page 5
Binding constant determination by competition experiment with EDTA Page 5
Selectivity studies by UV-Vis and ESI-MS Page 6-7
Cu^{2+} extraction from copper containing protein metallothionein-2 by **TB** Page 8-9
Full UV-Vis spectra for kinetics of ascorbic consumption experiments Page 10
References Page 10

ESI-MS and HPLC data of peptoid oligomer TB

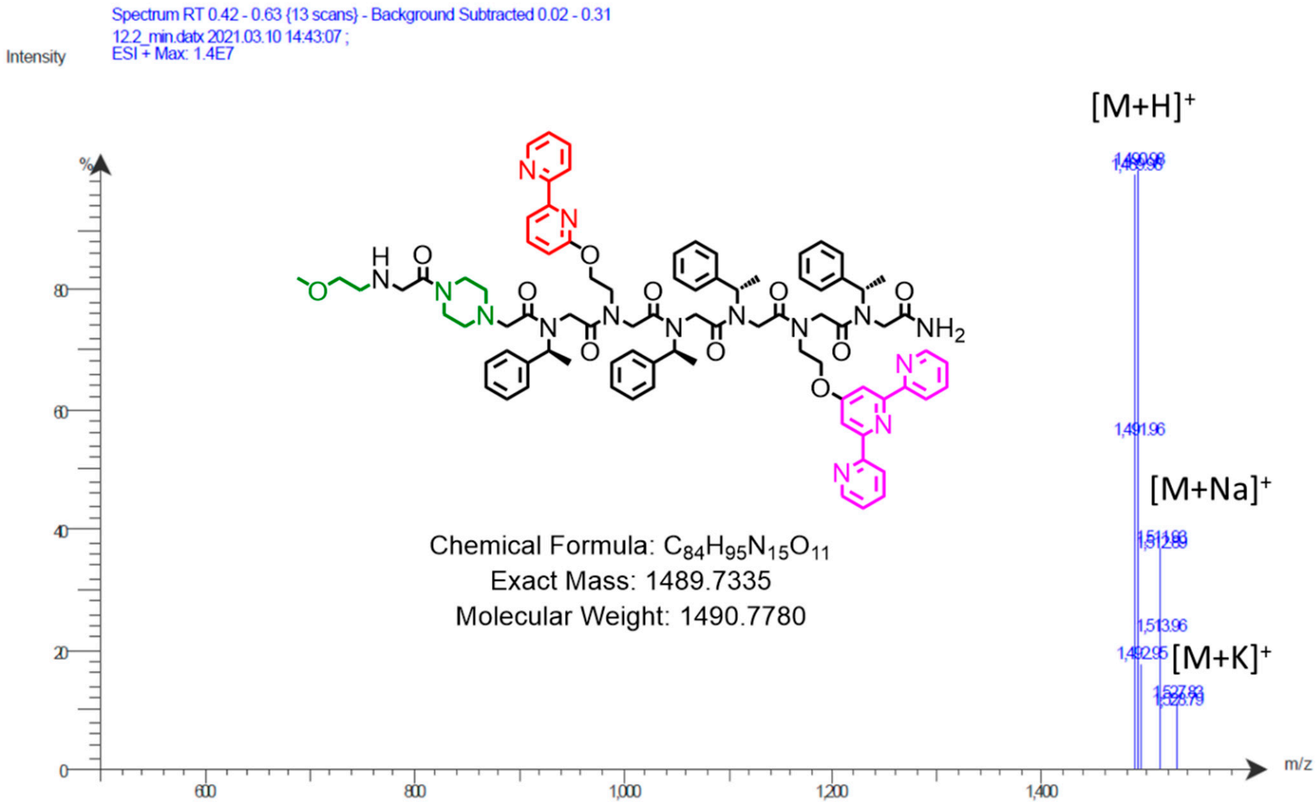

Figure S1 ESI-MS spectra of peptoid oligomer **TB** in acetonitrile

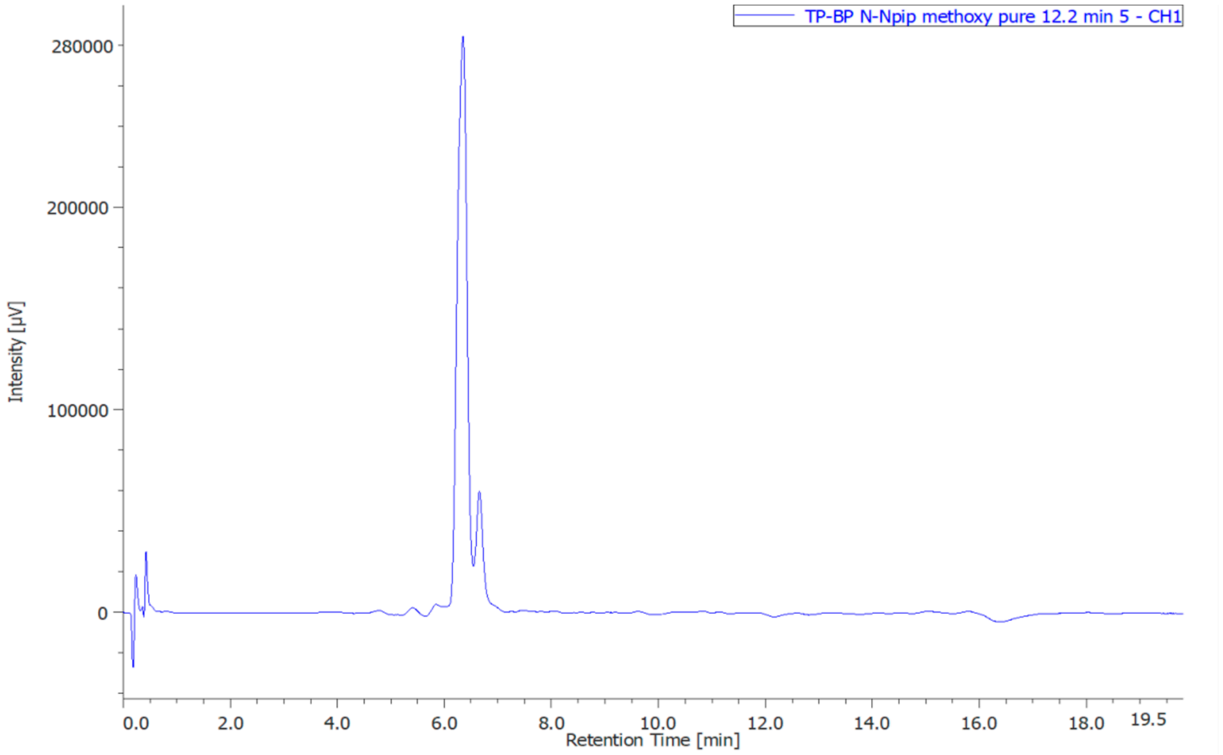

Figure S2 HPLC spectra of peptoid oligomer **TB** in acetonitrile

UV-Vis titrations with metals

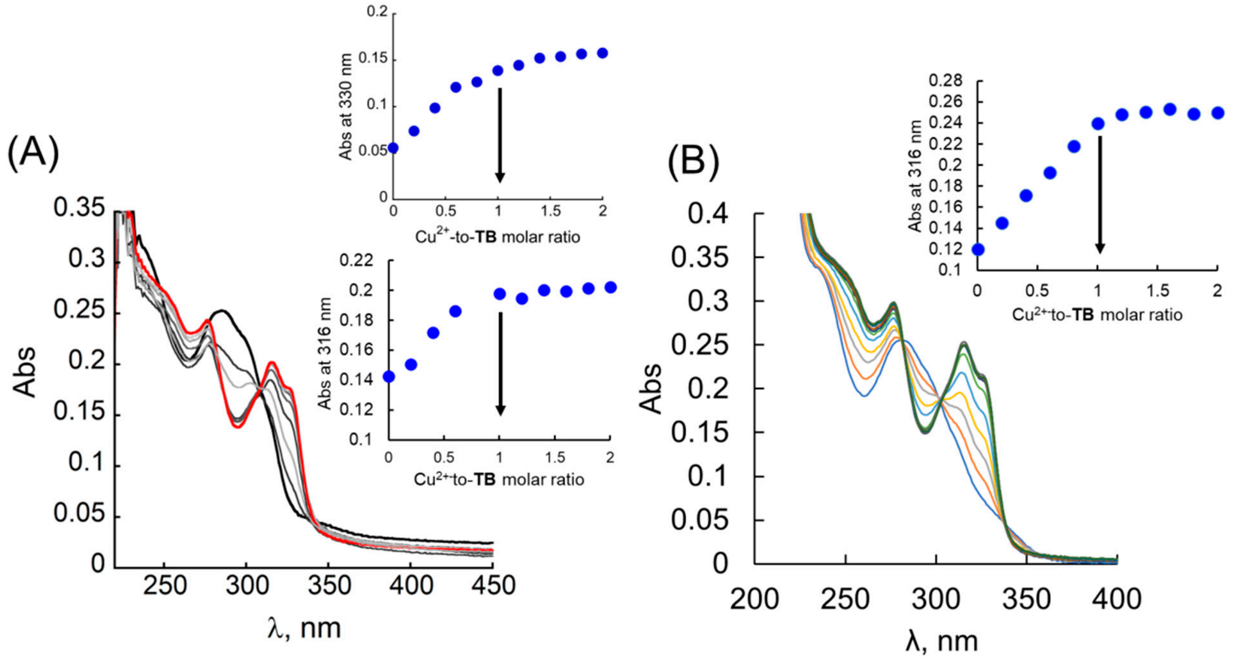

Figure S3 UV-Vis titration of **TB** (10 μM) with Cu^{2+} in (A) HEPES buffer (50 mM, pH=7.4). (B) un-buffered water (pH = 7.0) Inset: metal-to-peptoid ratio plots, constructed from the corresponding UV-Vis titration

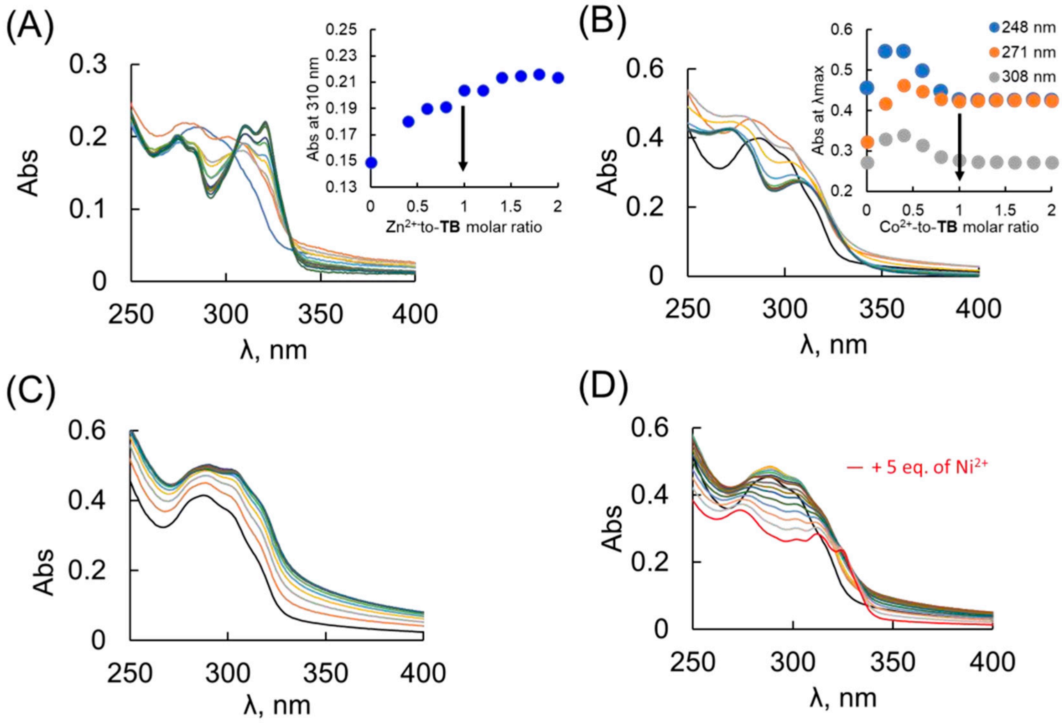

Figure S4 UV-Vis titration of **TB** (10-20 μM) with (A) Zn^{2+} (B) Co^{2+} (C) Mn^{2+} (D) Ni^{2+} in HEPES buffer (50 mM, pH=7.4). Insets: metal-to-peptoid ratio plots, constructed from the corresponding UV-Vis titration

HR ESI-MS data for Cu^{2+} complex with TB

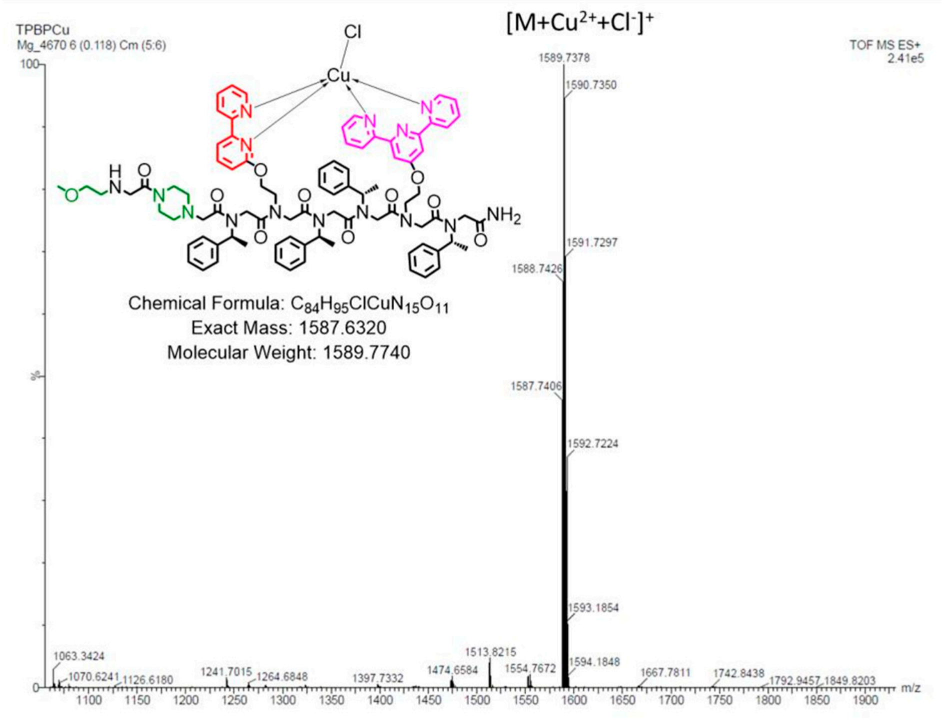

Figure S5 ESI-MS traces of mixture of 1 equiv. of **TB** with 1 equiv. of Cu^{2+} in HEPES buffer (50 mM, pH=7.4), suggesting formation of 1:1 Cu**TB** complex. Coordination of Cl^- is plausible as copper(II) chloride was used as a precursor salt for complexation.

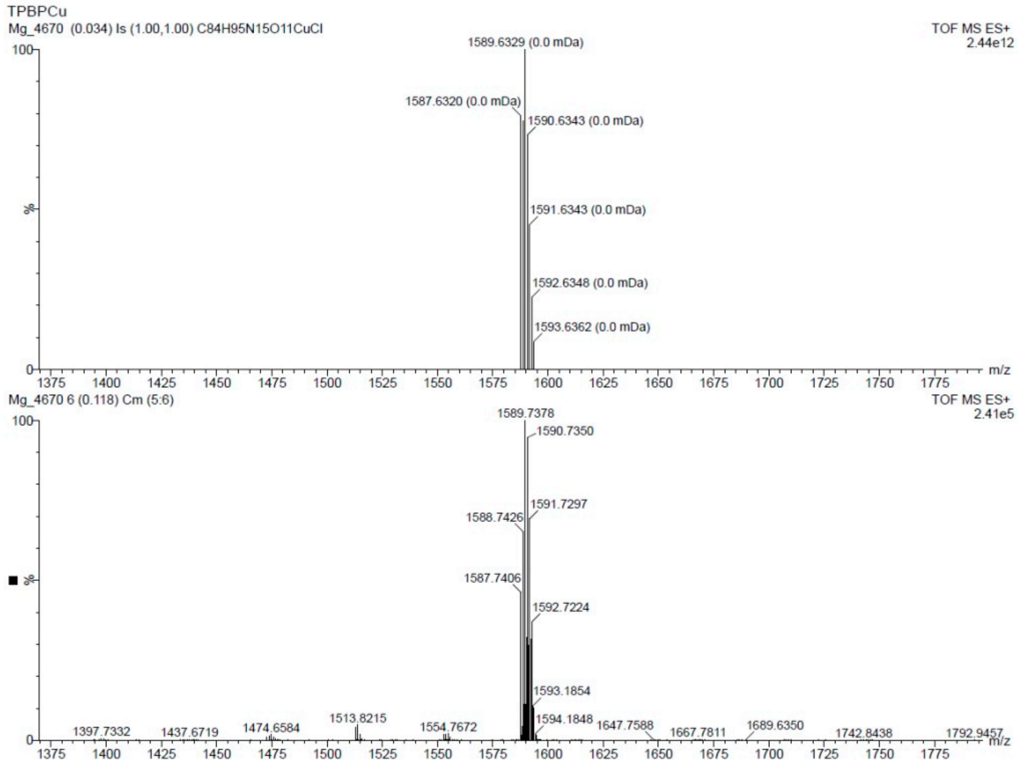

Figure S6 ESI-MS m/z traces of Cu**TB** (bottom) and calculated ESI-MS spectrum (top).

EPR data

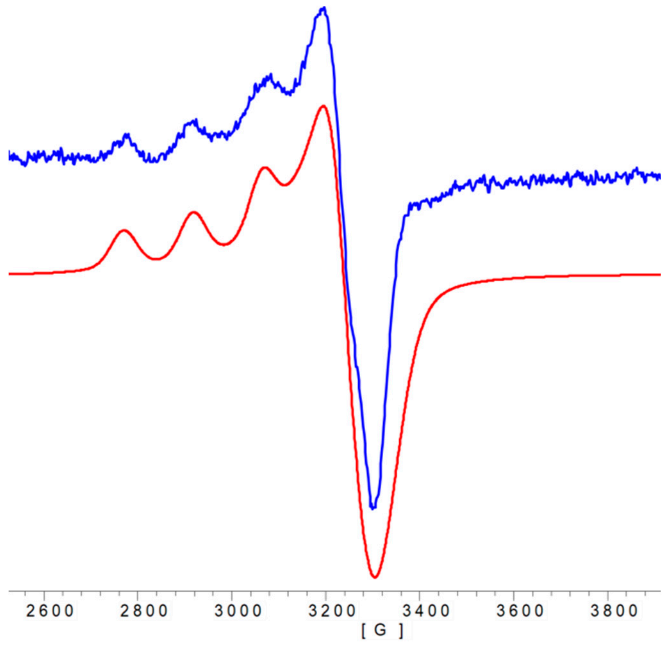

Figure S7. X-band EPR spectra of peptoid copper Cu**TB** complex (1 mM) in frozen solution state in HEPES (50 mM, pH = 7.4) buffer (blue line) and the corresponding simulated spectra (red line) measured at 203 K. Reference- (2,2,6,6-Tetramethyl-1-piperidinyl)oxidanyl (TEMPO, g = 2.0058).

Binding constant determination by competition experiment with EDTA

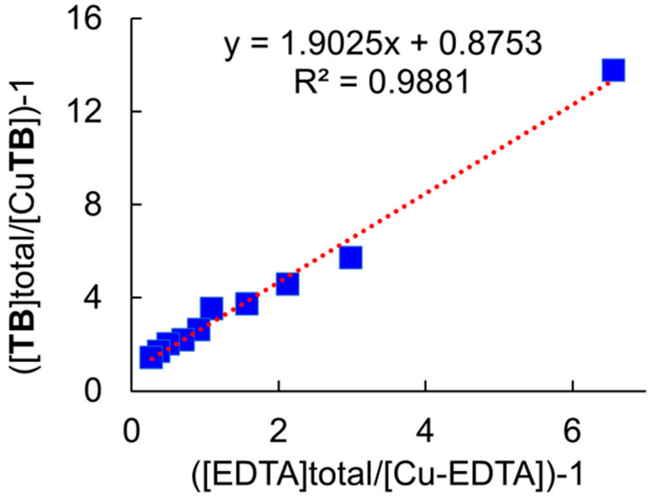

Figure S8. Binding affinity determination by competition method with EDTA. [1,2] The experiment has been executed in pH = 7.0, using EDTA as competitor agent. $CuSO_4$ is used as a metal ions source in the experiment. The formation constant for EDTA should be corrected for EDTA's acid–base properties in pH 7, which could be done by calculating the fraction, α(EDTA) [3]

Dissociation constant calculation for Cu**TB**:

Slope = K_D(Cu^{2+}-**TB**) ·K_A(Cu^{2+} -EDTA)· α(EDTA), for Cu^{2+}-**TB** is 6.28 x 10^{-16} M

[K_D: Dissociation constant of Cu^{2+}-**TB** complex, K_A: Association constant of Cu^{2+}-EDTA (6.309 x 10^{18} M^{-1}), and α(EDTA) is the pH correction factor].

Selectivity studies by UV-Vis and ESI-MS

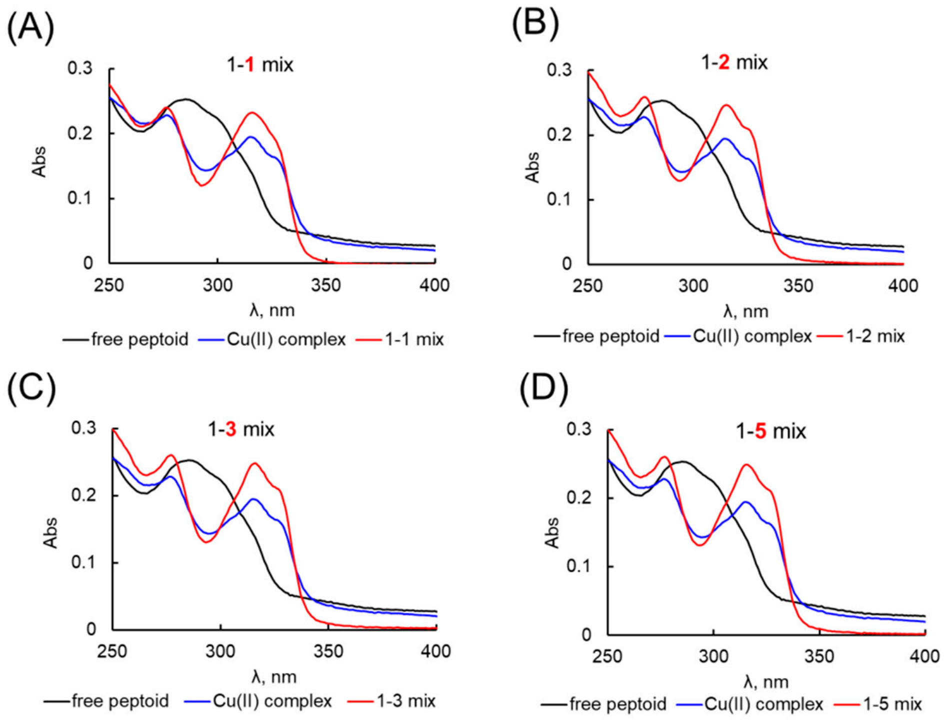

Figure S9. UV-Vis spectra of **TB** (17 μM, black), their Cu^{2+} complexes (10 μM, red) and the complexes formed upon mixing of 1 equiv. of **TB** with 1 equiv. of Cu^{2+} and (A) 1 equiv. of each Co^{2+}, Ni^{2+}, Mn^{2+}, Zn^{2+} (B) 2 equiv. of each Co^{2+}, Ni^{2+}, Mn^{2+}, Zn^{2+} (C) 3 equiv. of each Co^{2+}, Ni^{2+}, Mn^{2+}, Zn^{2+} (D) 5 equiv. of each Co^{2+}, Ni^{2+}, Mn^{2+}, Zn^{2+} in HEPES buffer, 50 mM

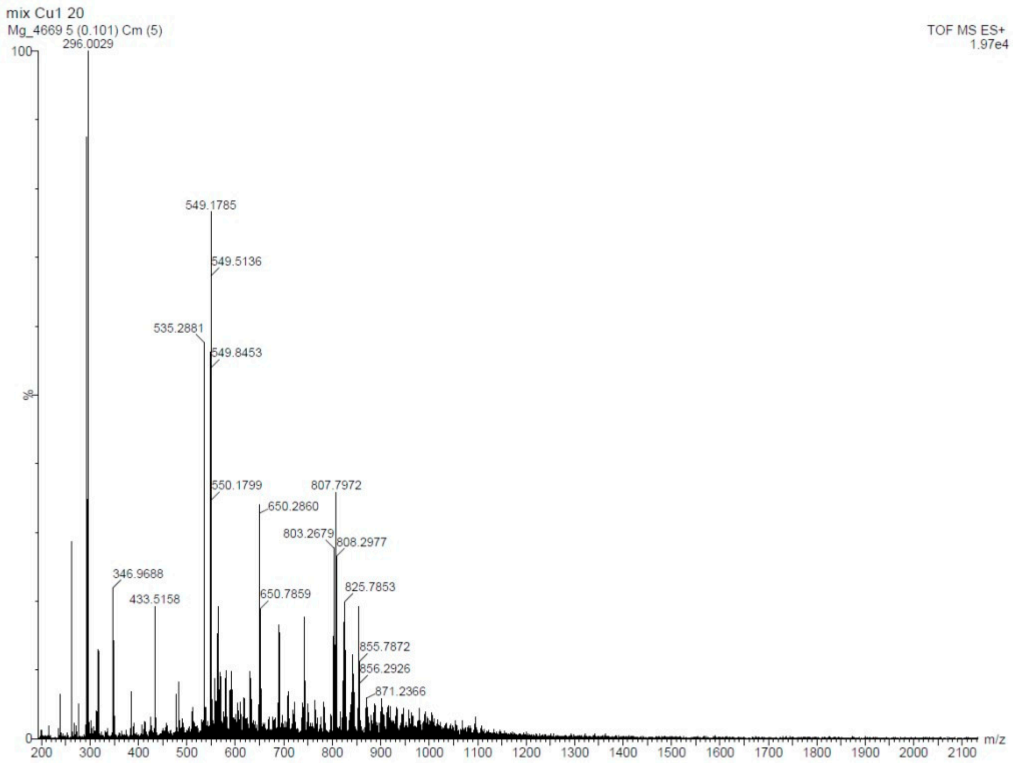

Figure S10. ESI-MS traces of the mixture of1 equiv. of peptoid oligomer **TB** with 1 equiv. of Cu^{2+} and 20 equiv. of each Co2+, Ni2+, Mn2+, Zn2+ in HEPES buffer, 50 mM pH = 7.4.

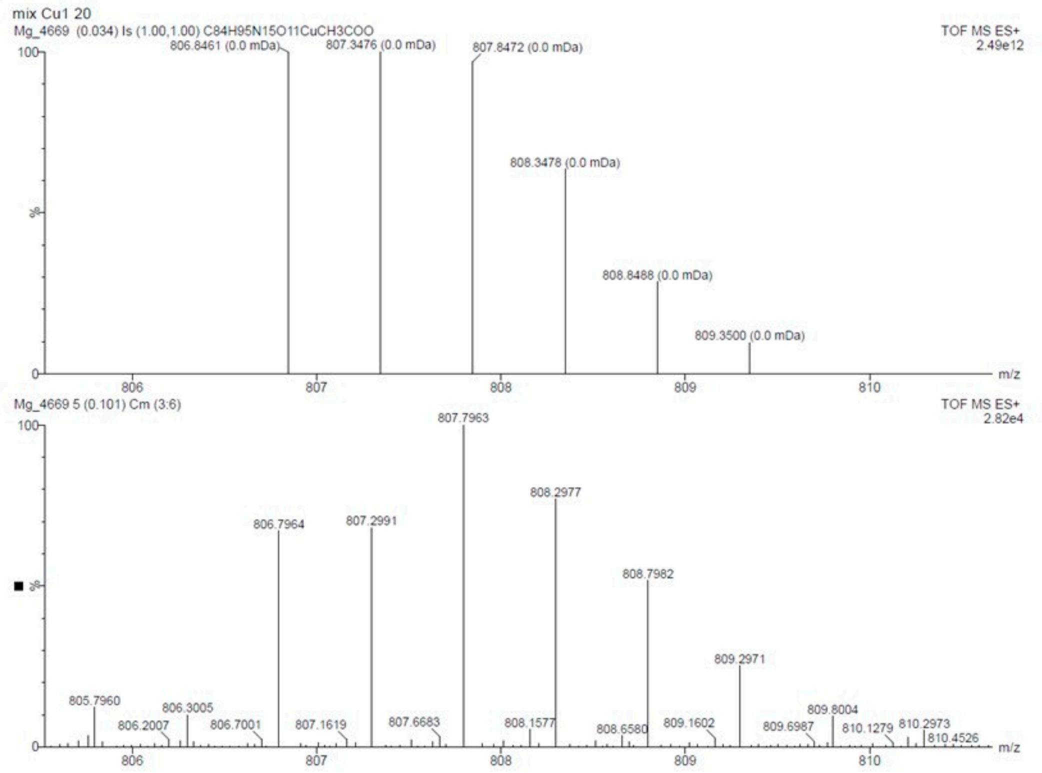

Figure S11 Experimental isotopic analysis by ESI-MS of Cu**TB**-acetate complex (bottom) and calculated ESI-MS spectrum (top) formed in a mixture solution of 1 equiv. of peptoid oligomer **TB** with 1 equiv. of Cu^{2+} and 20 equiv. of Co^{2+}, Ni^{2+}, Mn^{2+}, Zn^{2+} in HEPES buffer, 50 mM pH = 7.4.

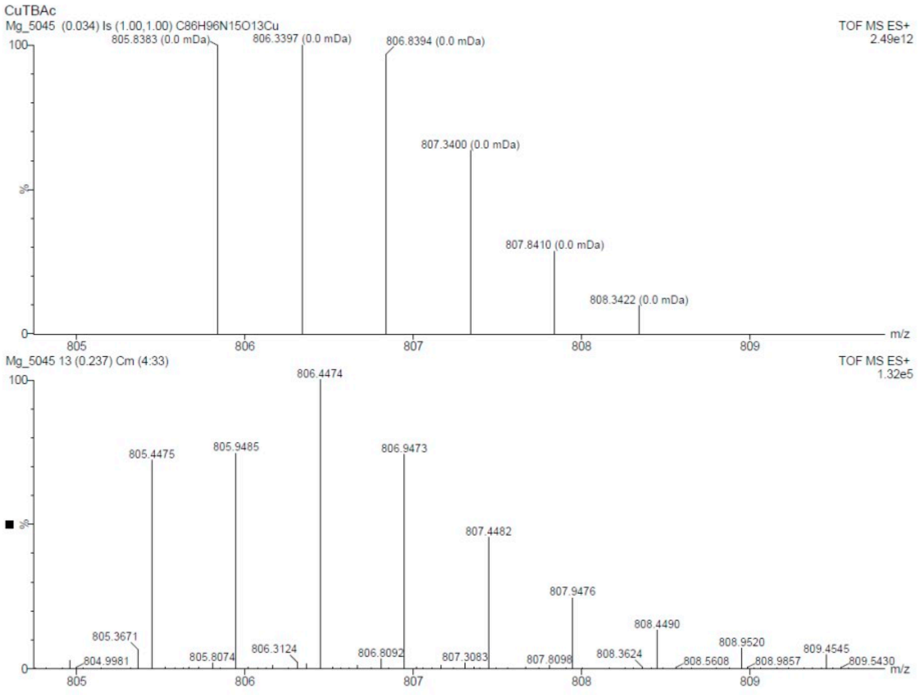

Figure S12 Experimental isotopic analysis by ESI-MS of Cu**TB**-acetate complex (bottom) and calculated ESI-MS spectrum (top), formed in a mixture solution of 1 equiv. of peptoid oligomer **TB** with 1 equiv. of Cu^{2+} (from Cu(II) acetate ion 61 source) in HEPES buffer, 50 mM pH = 7.4.

Cu^{2+} extraction from copper containing protein metallothionein-2 by TB

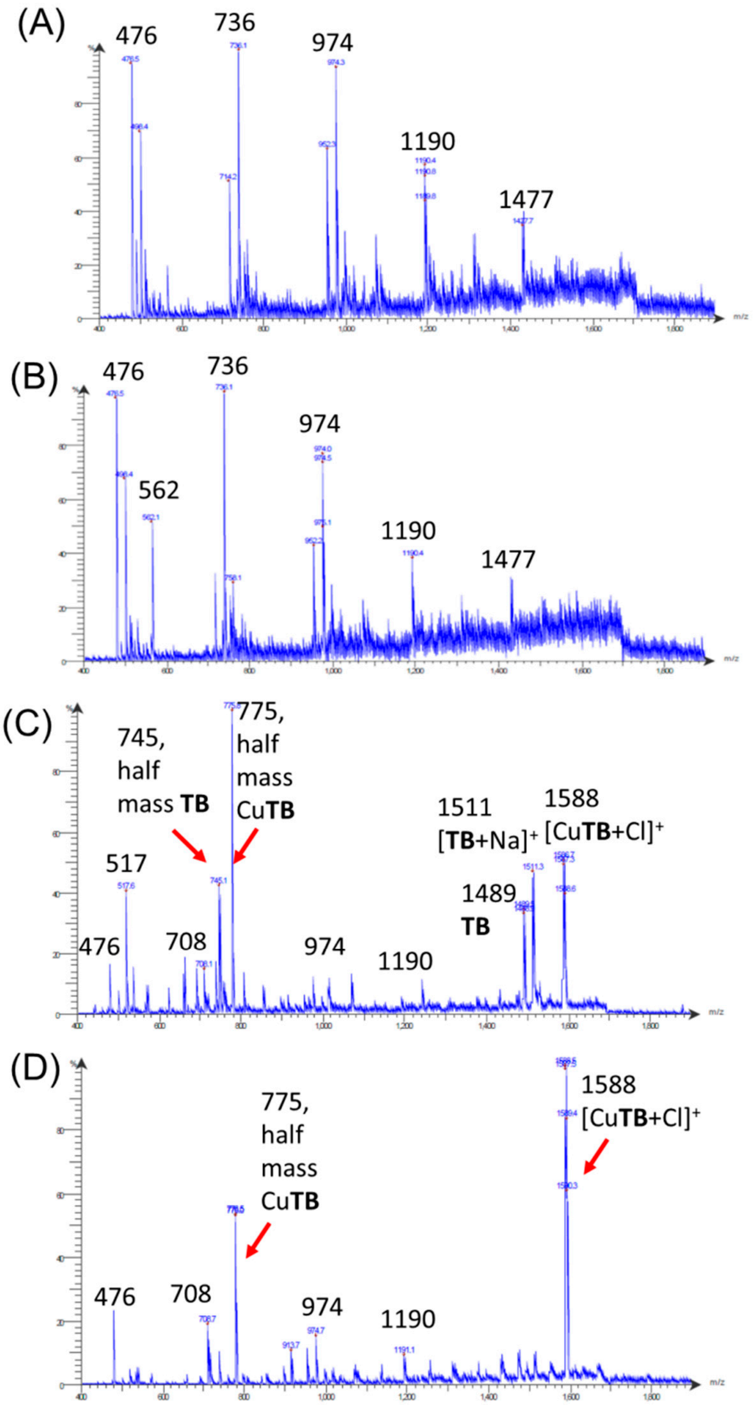

Figure S13 ESI-MS studies of the CD experiments depicted in Fig. 3C-D of the main manuscript text. (A) free MT-2 (B) MT-2 + 6 equiv. of Cu^{2+} . (C-D) mixture of MT-2 + Cu^{2+} + **TB** at (C) 30 min or (D) 12 hours after addition of **TB**. Conditions for (C): [MT-2] = 25 μM, [Cu^{2+}]=[**TB**]=150 μM for (D): [MT-2] = 33 μM, [Cu^{2+}]=[**TB**]=200 μM. For (A-D) HEPES buffer 10 mM pH = 7.4, excess of TCEP, 25 °C.

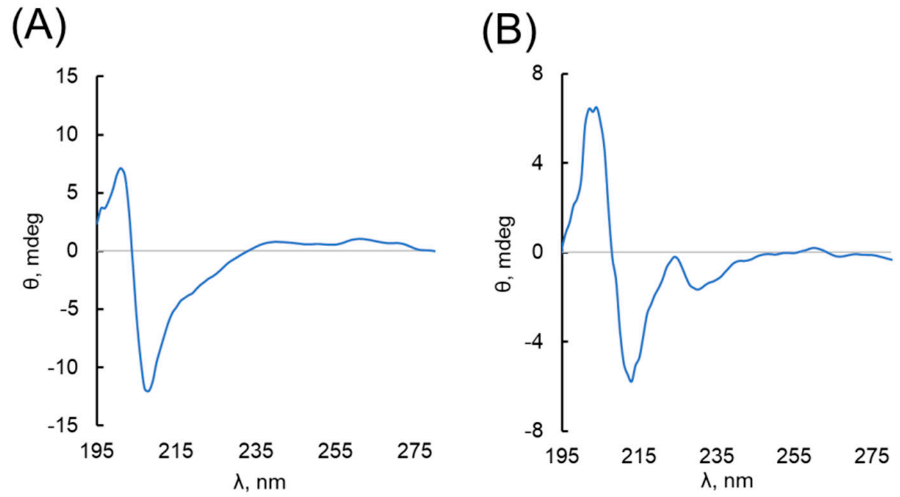

Figure S14 The CD spectra obtained by substraction of CD spectrum of Cu**TB** from the CD spectrum of the mixture of MT-2 + Cu^{2+} + **TB** at (A) 30 min and (B) 12 hours after addition of **TB.** Conditions for (A): [MT-2] = 25 µM, [Cu^{2+}]=[**TB**]=150 µM for (B): [MT-2] = 33 µM, [Cu^{2+}]=[**TB**]=200 µM. For (A-B) HEPES buffer 10 mM pH = 7.4, excess of TCEP, 25 °C.

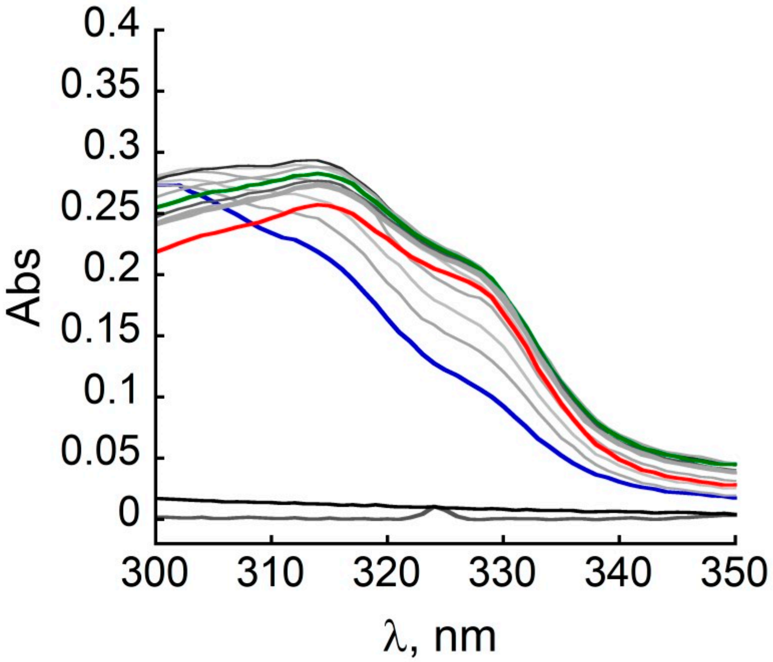

Figure S15. UV/Vis spectra in the near UV range of the kinetics of Cu^{2+} extraction from CuMT-2 by **TB.** Spectra were recorded every 30 sec, duration of experiment – 1 hour. For the sake of clarity, herein represented only spectra for every 300 sec after the addition of **TB**. MT-2 and CuMT-2 do not absorb in the near UV range (grey and dark grey curves at around 0 Abs). CuMT-2 + **TB** 0 sec after addition (blue), 1000 sec after addition (dark grey), 1800 sec after addition (green), 3600 sec after addition (red). Conditions: [MT-2] = 1.6 µM, [Cu^{2+}] = 9 µM, [**TB**] = 10 µM, in HEPES buffer (10 mM, pH = 7.4 with an excess of TCEP.

Full UV-Vis spectra for kinetics of ascorbic consumption experiments

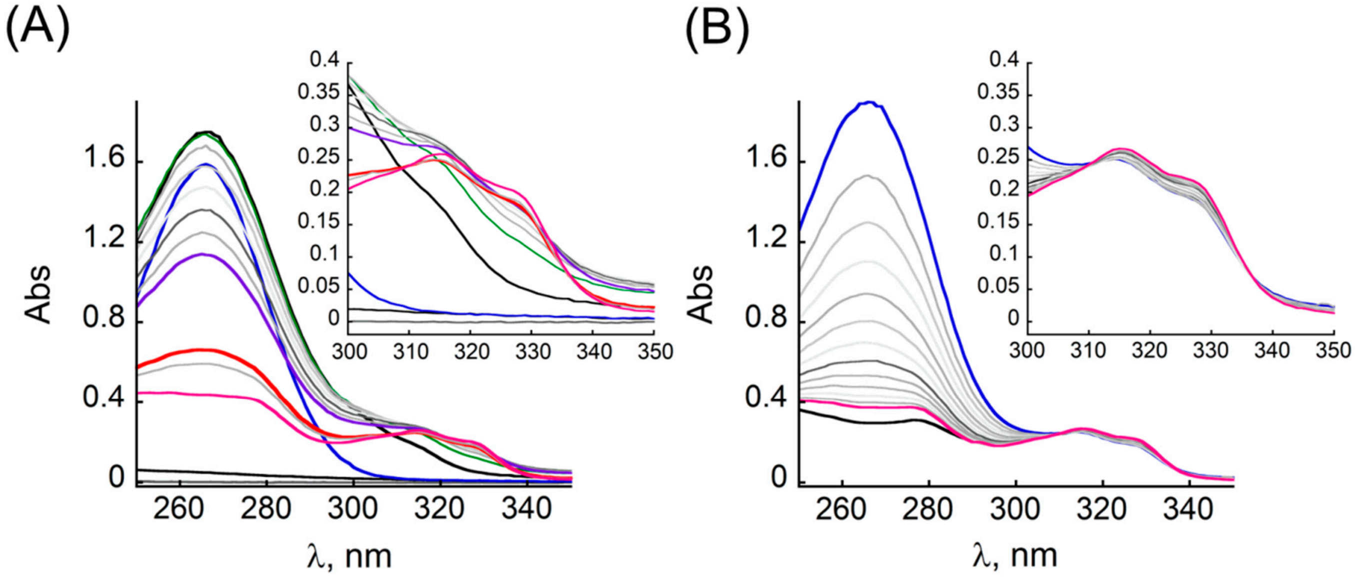

Figure S16. UV-Vis spectra of the kinetics of ascorbic consumption for (A) MT-2 + Cu^{2+} + Asc + **TB** (Fig. 6B, green). UV-Vis spectra depicted herein are as follows: MT-2 + Cu^{2+} + Asc (blue), MT-2 + Cu^{2+} + Asc + **TB** at 0 sec (black), MT-2 + Cu^{2+} + Asc + **TB** at 300 sec (green), MT-2 + Cu^{2+} + Asc + **TB** at 2070 sec (purple), MT-2 + Cu^{2+} + Asc + **TB** at 2100 sec (red), MT- 2 + Cu^{2+} + Asc + **TB** at 3600 sec (last spectrum, pink). (B) MT-2 + Cu^{2+} + **TB** (1 hour) + Asc (Fig. 6B, blue). UV-Vis spectra depicted here are as follows: MT-2 + Cu^{2+} + **TB** (1 hour) (black), MT-2 + Cu^{2+} + **TB** (1 hour) + Asc at 0 sec (blue), MT-2 + Cu^{2+} + **TB** (1 hour) + Asc at 3600 sec (pink). Grey curves show the dynamic of changes in the absorbance spectra for every 300 sec. Conditions: [MT-2] = 1.6 μM, [Cu^{2+}]= 9 μM [**TB**]=10 μM, [Asc] = 100 μM, in HEPES buffer 10 mM pH = 7.4.

References:

[1] Xiao, Z.; Wedd, A. G. The challenges of determining metal–protein affinities. *Nat. Prod. Rep.*, **2010**, *27*, 768 – 789.

[2] Zhang, L.; Koay, M.; Maher, M. J. ; Xiao, Z.; Wedd, A. G. Intermolecular transfer of copper ions from the CopC protein of Pseudomonas syringae. Crystal structures of fully loaded Cu(I)Cu(II) forms. *J. Am. Chem. Soc.*, **2006**, *128*, 5834 – 5850.

[3] Harvey, D.; *Modern Analytical Chemistry*, Wiley: New York, USA, 2000; p. 316
